# Influence of Electrohydrodynamics (EHD) on the Drying Characteristics and Active Ingredients of Astragalus Membranaceus Slices

**DOI:** 10.3390/foods14223935

**Published:** 2025-11-17

**Authors:** Ni Lan, Yongping Wang, Jingcheng Zhu

**Affiliations:** College of Science, Inner Mongolia University of Technology, Hohhot 010080, China; 202320908036@imut.edu.cn (N.L.); 202320908027@imut.edu.cn (Y.W.)

**Keywords:** electrohydrodynamic drying, medicinal herbs, ion wind, drying kinetics, effective constituent

## Abstract

This study compared needle-plate electrohydrodynamic drying (EHD) at 20, 25, and 30 kV to natural drying (ND) of Astragalus membranaceus slices, analyzing drying characteristics, quality, and mechanisms. Discharge diagnostics revealed filamentous discharge, with reactive nitrogen/oxygen species concentration and ion wind speed increasing with voltage. Within the 20–30 kV range, drying rate and effective moisture diffusivity significantly increased with electric field strength. At 30 kV, drying rate was 1.73 times ND’s, and diffusivity was 5.1 times higher. Quality was optimal at 25 kV: rehydration rate was 1.18 times ND’s; calycosin and astragaloside IV contents were 1.38 and 1.14 times ND’s, respectively; shrinkage was reduced to 0.68 times ND’s; and browning was significantly inhibited (BI = 0.46 times ND’s), yielding the color closest to fresh samples. Polysaccharide content was slightly lower (0.97 times ND’s). In summary, EHD, particularly at 25 kV, markedly enhances drying efficiency and improves key quality attributes (rehydration, bioactive compound retention, color, reduced shrinkage), despite a minor negative effect on polysaccharides. This work clarifies the EHD mechanism and supports its application in drying traditional Chinese medicines.

## 1. Introduction

Astragalus membranaceus, a medicinal and edible herb, not only has a long-standing application history in traditional medicine but also exhibits extensive potential in modern medical applications. It holds significant research and development value and has been attracting increasing international attention. Astragalus is widely used in the fields of pharmaceutical development, catering, and health care. The market demand is continuously increasing, with an annual export volume of 2000 tons [1]. This herb contains abundant trace elements and bioactive compounds, imparting pharmacological properties including immunomodulation, antitumor activity, qi-invigoration, spleen-strengthening, and anti-aging effects. With the implementation of the Healthy China Initiative, the market demand for traditional Chinese medicinal materials like Astragalus membranaceus has been on an upward trajectory. However, upon harvest, Astragalus membranaceus typically exhibits a high Moisture content (wet basis) ranging from 40% to 60%. Furthermore, its pronounced thermosensitivity and hydrophilicity predispose it to mildew and decay. Consequently, it is essential to subject Astragalus membranaceus to drying processes to reduce its moisture content and thereby preserve its quality and medicinal efficacy [2,3,4,5].

At present, in the place of origin of astragalus, the natural drying method of slicing the astragalus into thin pieces and laying them flat on the ground is commonly used. Although this method is energy-efficient, it suffers from several drawbacks, including a protracted drying cycle, substantial land occupation, and high vulnerability to environmental fluctuations. Additionally, the quality of the dried products is often suboptimal, primarily due to uncontrolled environmental conditions leading to dust contamination, microbial growth, uneven drying, and potential rain damage. Other alternative drying methods also present one or more limitations. For instance, freeze-drying can effectively preserve the active ingredients of Astragalus membranaceus; nevertheless, it is characterized by high energy consumption and a long drying duration. Hot air drying can rapidly dehydrate the material, yet it consumes a large amount of energy, and the high temperatures during the drying process may trigger thermal decomposition of certain components in Astragalus membranaceus, which is detrimental to the retention of its active ingredients. Microwave vacuum drying can maintain the color of the dried product relatively well, but it may cause surface collapse of the product. Additionally, the associated drying equipment is prohibitively expensive, thereby precluding its large-scale application [6,7].

In this study, electrohydrodynamic (EHD) drying technology was employed. This technology features simplicity in operation. During the drying process, an ion wind is generated between the emission electrode and the ground electrode. This ion wind interacts with the water molecules within the material [8]. Water molecules migrate toward the region of higher electric field intensity under the influence of the electric field force [9]. This directional movement of water molecules facilitates the removal of moisture from the material. Critically, EHD is a non-thermal process, rendering it energy-efficient and eco-friendly. Moreover, it helps to retain the nutritional components of the material. Additionally, this technology can enhance the sensory quality of traditional Chinese medicine products, thereby contributing to the improvement of the industry’s economic efficiency [10]. In the existing studies, high-voltage electric field drying (HVEFD), plasma drying (PD), and electrohydrodynamic drying (EHD) are classified as the same type of drying methods [11]. Said type has multiple advantages such as energy saving, sterilization, and improvement of the quality of dried products [12].To facilitate the integration of traditional and modern technologies in the processing of Chinese medicinal materials, the promotion of novel drying technologies is of great urgency. Recently, the potential of electrohydrodynamic (EHD) principles for drying Astragalus membranaceus slices was demonstrated by Hou et al. using HAPD, CPD (EHD), PD, ND and HAD. However, the effects and mechanisms of standalone EHD drying, without synergistic airflow, remain unexplored [13].

Previously, several scholars have applied EHD technology to the drying of various materials. Peng Guan et al. compare the effects of EHD, hot air drying, and natural air drying on the physicochemical properties of Pleurotus ostreatus. Their findings indicate that EHD at 27 kV results in a superior drying rate and better quality of Pleurotus ostreatus [14]. In a separate investigation, Jie Zhu et al. evaluated the impact of EHD on the drying characteristics of Zingiber officinale, reporting a significant acceleration in drying rate with minimal aldehyde formation observed at 30 kV [15]. However, to date, no research has been conducted specifically on the application of single-mode EHD to Astragalus membranaceus slices.

Therefore, the objectives of this study were to complete the following:

(1) By diagnosing the discharge characteristics of EHD (including the detection of ion wind, the measurement of voltage and current waveforms, and the determination of the types and concentrations of plasma generated during discharge), the drying mechanism of EHD for drying astragalus slices was studied.
(2) Through conducting drying experiments, the drying characteristics of Astragalus membranaceus slices under the action of EHD (including the trends of moisture content and moisture ratio over time, the trends of drying rate over time, the effective moisture diffusion coefficient, etc.) were studied and compared with the drying characteristics of the traditional drying method—natural drying. Comprehensive data on the drying characteristics of EHD on Astragalus membranaceus slices were obtained.
(3) By measuring and analyzing the rehydration rate, shrinkage rate, surface color, and the retention of nutritional components such as astragaloside in the Astragalus membranaceus slices after drying, the impact of different drying methods on the quality of Astragalus membranaceus slices was evaluated.

It is hoped that through these experimental goals, the drying and storage quality of astragalus can be improved, and at the same time, that this study will provide an experimental basis and practical guidance for the wide application of electro-fluid dynamics drying technology.

## 2. Materials and Methods

### 2.1. Experimental Materials

This study selected astragalus from Baian Nur City, Inner Mongolia as the experimental material. After screening, uniform-sized astragalus roots with a diameter of 10 ± 2 mm and a length of 30 ± 10 cm were selected. The roots were washed under running tap water for 2 min to remove surface soil and impurities. Subsequently, they were cut into 2 mm-thick slices using a commercial stainless steel vegetable slicer (0702QTZD, MYTEC, Linyi, China). The rapid moisture analyzer (SH10A, Shanghai, China, with a precision of ±0.1%) was employed for its speed and operational convenience in rapidly determining the initial moisture content for experimental setup, which was crucial for ensuring the consistent initial state across all samples. To validate its accuracy, the readings were cross-verified against the standard oven method (drying at 105 °C for 24 h) in preliminary tests. A paired *t*-test showed no significant difference between the results obtained from the two methods (*p* > 0.05). The initial moisture content was measured via a rapid moisture analyzer (SH10A, Shanghai, China, with a precision of ±0.1%) and determined to be 53.8% ± 0.5% (w.b.). During the drying experiment, the weight of the astragalus slices was measured every 30 min using an electronic balance (BS124S, Shanghai Guanglu Electronic Technology Co., Ltd. (Shanghai, China)) until the weight reached was unchanged, which was defined as a mass loss of less than 0.01 g between two consecutive measurements, at which point the experiment was terminated. A fixed weighing interval was used to maintain operational consistency and ensure synchronous data collection across all treatment groups.

### 2.2. Experimental Instruments and Equipment

**Figure 1:**
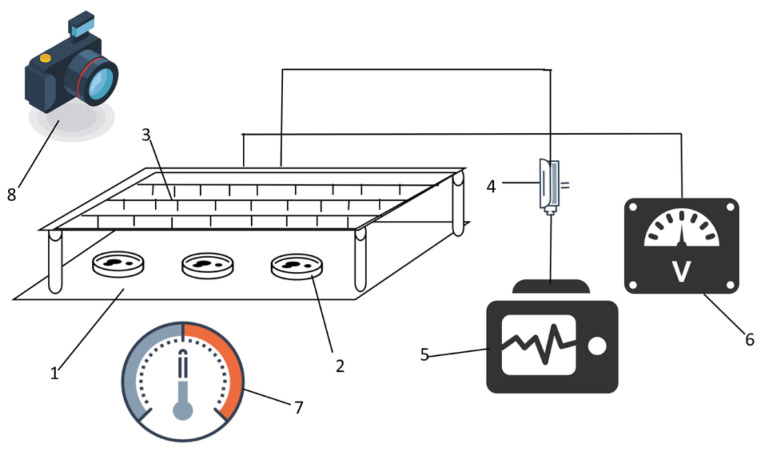
Schematic diagram of EHD device. 1. Bottom crown. 2. Sample. 3. Needle electrode. 4. Current Probe. 5. Oscilloscope. 6. Voltage control system. 7. Thermometer-hygrometer. 8. Camera.

As shown in Figure 1, the fluid dynamics drying device mainly consists of the YD(JZ)-1.5/50 type high-voltage power supply (Wuhan, China), the KZX-1.5 kVA type controller, and the multi-pin-board electrode system. The high-voltage power supply is used to output alternating current voltage, and its controller has the ability to adjust the alternating current voltage, with the voltage range covering 0 to 50 kV. The electrode system consists of multi-pin electrodes of size 75 cm × 28 cm, totaling 98 needle-shaped electrodes. The distance between the needle-shaped electrodes is 40 mm, each electrode has a diameter of 1 mm, a length of 20 mm, a curvature radius of the needle tip of 0.5 mm, and a needle tip length of 1 mm. The lower electrode plate of the electrode system is made of a 100 cm × 45 cm stainless steel plate, with a needle-board spacing of 8 cm. The electronic balance, the constant temperature water bath pot, and the automatic colorimeter (3nh-NR60CP, Shenzhen, China) are also included.

### 2.3. Experimental Scheme

The prepared 2 mm-thick, uniformly thickened astragalus slices were placed in a Petri dish and placed on the lower electrode plate for single-factor experiments. By changing the electric field voltage: 20 kv, 25 kv, 30 kv, the optimal parameters were explored, and a set of natural air-drying (ND) was set as the control group. The ND samples were placed in a shaded, well-ventilated laboratory environment at 20 °C ± 2 °C and 26% ± 2% RH, wind speed: 0.10 ± 0.01 m per second avoiding direct sunlight and dust, to simulate a controlled shade-drying condition. The mass of the astragalus slices for both EHD and natural drying (ND) groups was measured every 30 min using the electronic balance (BS124S, Shanghai Guanglu Electronic Technology Co., Ltd. (Shanghai, China)) until the weight remained unchanged (the weight was measured twice in a row, and the change in weight was less than 0.01 each time), at which point the experiment was terminated. To reduce experimental errors, each group of experiments was repeated three times and the average value was taken. By adjusting the EHD voltage parameters (20 kV, 25 kV, 30 kV), the effects of different parameters on the emission spectrum, ion wind speed, discharge morphology, and volt-ampere characteristics were explored.

### 2.4. Index Measurement and Methods

#### 2.4.1. Measurement of Ion Velocity

Ion wind velocity under varying discharge voltages was quantified using a calibrated thermal anemometer probe (Testo 4051, Titisee-Neustadt, Germany). Measurements were conducted axially beneath the needle electrode, with the probe maintained at a fixed distance of 8 cm from the needle tip. Triplicate measurements were performed for each voltage condition, with results expressed as mean ± standard deviation.

#### 2.4.2. Measurement of Voltage–Current Waveforms and Discharge Morphology

The voltage and current waveforms generated by the plasma drying system were captured via a configuration comprising an oscilloscope, a high-voltage probe, and a current detector (Siglent Company from Shenzhen, China). The applied discharge voltages were 20 kv, 25 kv, and 30 kv, with a frequency of 50 Hz alternating current. For voltage measurement, the high-voltage probe was arranged with its sensing end at the needle electrode and its ground terminal connected to the grounding plate, thereby establishing a parallel circuit. The grounding wire passed through the circular hole of the current monitor. The high-voltage probe and the current monitor were connected to the oscilloscope to obtain the voltage and current waveform diagrams of discharge under different voltages. The determination of discharge morphology was carried out in a dark environment, using a single-lens reflex camera to photograph the discharge process under different voltages, obtaining the discharge morphology under the action of different voltages.

#### 2.4.3. Determination of Plasma Emission Spectra

The discharge process under different discharge voltages was measured using an intensified charge-coupled device (ICCD) (DH334T-18 U-E3, Belfast, UK), and emission spectra within the wavelength range of 200–1000 cm^−1^ were obtained. This enabled the diagnosis of the particle types and concentration changes in the plasma produced by different discharge voltages.

#### 2.4.4. Determination of Dry Base Moisture Content

The initial moisture content was a%, and $m_{0}$ represents the mass of astragalus at time 0.

The formula for calculating dry base moisture content [13] is as follows:

(1) $$M_{t}=\frac{m_{t}-m_{g}}{m_{g}}$$

where $M_{t}$ is the dry base moisture content of the sample at time t (unit: g/g); $m_{t}$ is the mass of the astragalus slice at time t (unit: g); and $m_{g}$ is the dry mass of the astragalus slice (unit: g).

(2) $$m_{g}=m_{0}\times \left(1-a\%\right)$$

#### 2.4.5. Determination of Moisture Ratio

To more intuitively reflect the drying degree and drying speed of the material, we measured the moisture ratio of the astragalus slices. The calculation formula is as follows [16]:

(3) $$M_{R}=\frac{M_{t}-M_{e}}{M_{0}-M_{e}}$$

where $M_{e}$ is the dry base moisture content at equilibrium, g/g. Since $M_{e}$ is very small compared to $M_{t}$ and $M_{0}$, and the final moisture content of all samples was confirmed to be below 8.5% (d.b.), the calculation result is not significantly affected, so the calculation formula for the moisture ratio of the astragalus slices is simplified as follows:

(4) $$M_{R}=\frac{M_{t}}{M_{0}}$$

where $M_{0}$ is the initial dry base moisture content of the astragalus slice, g/g.

#### 2.4.6. Determination of Drying Rate

The drying rate can represent the evaporation rate of moisture in the material per unit time. The calculation formula is as follows [17]:

(5) $$D_{R}=\frac{M_{t}-M_{t+∆t}}{∆t}$$

where $D_{R}$ is the drying rate (unit:(g water/g solid/min), $M_{t}$ is the moisture content of the astragalus at time t, $M_{t+∆t}$ is the moisture content of the astragalus at time t+∆t.

#### 2.4.7. Determination of Effective Moisture Diffusivity

Based on Fick’s second law to describe the basic law of water diffusion, and considering the influence of temperature on the diffusion coefficient using the Arrhenius equation, the drying process of the astragalus slice is described as follows [18]:

(6) $$M_{R}=\frac{8}{\pi^{2}}\sum_{n=0}^{\infty }\frac{1}{(2n+1{)}^{2}}\exp\left(-\frac{(2n+1{)}^{2}\pi^{2}D_{eff}t}{4L^{2}}\right)$$

If the drying time is long enough for the $M_{R}<0.6$ (a common assumption in thin-layer drying models based on Fick’s second law), then the equation simplifies to the follwing:

(7) $$LnM_{R}=\ln\left(\frac{8}{\pi^{2}}\right)-\frac{\pi^{2}D_{eff}}{4L^{2}}t$$

The simplified equation shows a linear characteristic. Therefore, the relationship image between $\ln M_{R}$ and time t will be a straight line.

The slope $k_{0}$ of this straight line is associated with the effective water diffusion coefficient $D_{eff}$.

(8) $$k_{0}=-\frac{\pi^{2}D_{eff}}{4L^{2}}$$

(9) $$D_{eff}=-k_{0}\frac{4L^{2}}{\pi^{2}}$$

where $D_{eff}$ is the effective water diffusion coefficient, $m^{2}/s$; t is the fluid dynamics drying time, s; and L is half the thickness of the astragalus slice,  m.

Through the effective water diffusion coefficient $D_{eff}$, the diffusion characteristics of water in the drying process of the astragalus slice can be quantitatively described.

#### 2.4.8. Determination of Rehydration Rate

Rehydration rate is an important indicator for evaluating the quality of dried products, directly reflecting the ability of the dried products to absorb water and return to their original state. For rehydration, the dried astragalus slices were subjected to a 10 h immersion in a constant-temperature water bath maintained at 37 °C. After being removed from the water bath, the surface moisture was wiped dry with filter paper and the slices were allowed to rest for 5 min at room temperature to allow for surface moisture equilibration. Then, the mass of the astragalus before and after rehydration was measured using an electronic balance. The rehydration rate of astragalus slices can be calculated using the following formula [19]:

(10) $$R_{R}=\frac{m_{a}}{m_{b}}$$

where $R_{R}$ is the rehydration rate of astragalus, $m_{a}$ is the mass of astragalus after rehydration (unit: g), and $m_{b}$ is the mass of astragalus before rehydration (unit: g).

#### 2.4.9. Determination of Shrinkage Rate

To assess the effects of different drying parameters on the appearance and texture of astragalus slices, the shrinkage rate was determined using a volumetric displacement method. The volume of the fresh and dried astragalus slices was measured by dispersing dry sand in a graduated cylinder. The volume difference before and after sample immersion was recorded as the sample volume. The calculation formula is as follows [20]:

(11) $$S_{R}=\frac{V_{0}-V_{f}}{V_{0}}\times 100\%$$

where $S_{R}$ is the shrinkage rate of astragalus, $V_{0}$ is the volume of astragalus (unit: ${cm}^{3}$), and $V_{f}$ is the volume of dried astragalus (unit: ${cm}^{3}$).

#### 2.4.10. Determination of Surface Color Difference

The surface color difference can intuitively reflect the intensity of reactions such as oxidation and browning of Astragalus membranaceus slices during the drying process. Taking the color of a white reference board as the standard, the surface brightness value L*, redness value a*, and yellowness value b* of dried Astragalus membranaceus slices were measured using an automatic colorimeter (3nh-NR60CP, Shenzhen). When L*=0, it represents black; when L*=100, it represents white. A positive +a value indicates a red shift, a negative −a value indicates a green shift, a positive +b value indicates yellow, and a negative −b value indicates a blue shift. ∆E represents the color change value.

(12) $$∆E=\sqrt{∆L^{2}+∆a^{2}+∆b^{2}}$$

In the equation, ∆E represents the color difference value; $L_{0},a_{0},b_{0}$ represents the measured value of fresh Astragalus membranaceus; and L*,a*,b* represents the measured value of dried Astragalus membranaceus products.

(13) $$∆L=L*-L_{0}$$

(14) $$∆a=a*-a_{0}$$

(15) $$∆b=b*-b_{0}$$

CH represents chroma, which can reflect the saturation of the sample’s color. The larger the CH value, the brighter the sample’s color.

(16) $$CH=\sqrt{a*2+b*2}$$

BI represents the browning index, which can indicate the degree of browning in the sample. The larger the BI value, the more severe the browning of the sample [21].

(17) $$BI=\frac{100}{0.17}\left(\frac{a*+1.75L*}{5.645L*+a*-3.012b*}-0.31\right)$$

#### 2.4.11. Determination of Total Polysaccharides

The total polysaccharide content in the dried Astragalus membranaceus products was quantified employing the phenol-sulfuric acid method. The specific operation steps are as follows: accurately weigh an appropriate amount of standard glucose reference substance [22], add 1.0 mL of 6% phenol solution, and then add 5.0 mL of concentrated sulfuric acid. Shake well and cool. Let it stand at room temperature for 20 min and then measure the absorbance at a wavelength of 490 nm. Accurately weigh about 0.1 g of Astragalus membranaceus dry product powder that has been sieved, place it in a conical flask with a stopper, and accurately add 1.0 mL of 6% phenol solution and 5.0 mL of concentrated sulfuric acid. Shake well and cool. Let it stand at room temperature for 30 min. Then, accurately pipette 10 μg, 20 μg, 40 μg, 60 μg, 80 μg, and 100 μg of the reference solution and 0.1 mL of the test solution, and measure the absorbance at a wavelength of 490 nm. The results can then be obtained.

#### 2.4.12. Determination of Astragaloside IV

Astragaloside IV was determined by high performance liquid chromatography (HPLC) (General Chapter 0512) [23]. The specific operation steps are as follows: accurately weigh an appropriate amount of astragaloside IV reference substance and prepare a solution containing 0.5 mg per 1 mL with 80% methanol. Accurately weigh about 1 g of the sieved dry product of Astragalus membranaceus, place it in a conical flask with a stopper, accurately add 50 mL of 80% methanol solution containing 4% concentrated ammonia solution (take 4 mL of concentrated ammonia solution, add 80% methanol and dilute to 100 mL, shake well), seal it, weigh it, heat and reflux for 1 h, cool it down, and weigh it again. Make up the reduced weight with 80% methanol solution containing 4% concentrated ammonia solution, shake well, and filter. A 25 mL aliquot of the filtrate was accurately measured and concentrated to dryness. The resulting residue was then reconstituted in 80% methanol, quantitatively transferred to a 5 mL volumetric flask, and brought to volume with the same solvent. The solution was thoroughly mixed and filtered prior to analysis. Then, accurately take 5 μL and 10 μL of the reference substance solution and 10–20 μL of the test solution and inject them into the HPLC for determination. Calculate the content by the external standard two-point method logarithmic equation, and the result can be obtained.

Chromatographic conditions: Chromatographic column: Agilent, Shanghai, China, ZORBAX SB - C18 (4.6 × 250 mm, 5 μm); Detector: Evaporative Light Scattering Detector Flow rate: 0.8 mL/min; Column temperature: 35 °C; Mobile phase: Acetonitrile–Water (32:68).

#### 2.4.13. Determination of Calycosin

Calycosin was determined by high performance liquid chromatography (General Chapter 0512), and the specific operation steps were as follows: An appropriate amount of calycosin glucoside reference substance was accurately weighed and dissolved in methanol to prepare a solution containing 50 μg per 1 mL. About 1 g of the powder of Astragalus membranaceus dry products that had been sieved was accurately weighed and placed in a round bottom flask. A total of 50 mL of methanol was accurately added, and the weight was recorded. The mixture was heated and refluxed for 4 h, cooled, and the weight was recorded again. The reduced weight was made up with methanol, shaken well, and filtered. Then, 25 mL of the filtrate was accurately measured, and the solvent was evaporated to dryness. The residue was dissolved in methanol and transferred to a 5 mL volumetric flask. The volume was made up to the mark with methanol and shaken well. A total of 10 μL of the reference solution and the test solution were accurately injected into the liquid chromatograph for determination, and the results were obtained.

#### 2.4.14. Statistical Analysis

All experiments were conducted in triplicate to ensure methodological robustness and reproducibility. Primary data processing and organization were performed using Microsoft Excel 2016. Subsequent statistical analyses and graphical representations were generated using Origin 2024, including heat map visualizations and Pearson correlation matrices constructed via the Correlation Plot plugin. Intergroup comparisons were assessed through one-way analysis of variance (ANOVA) implemented in SPSS 22.0, with statistical significance established at *p* < 0.05. This integrated analytical framework ensured rigorous interpretation of experimental outcomes and supported the validity of derived conclusions.

## 3. Results and Discussion

### 3.1. Analysis of Discharge Characteristics of EHD System

**Figure 2:**
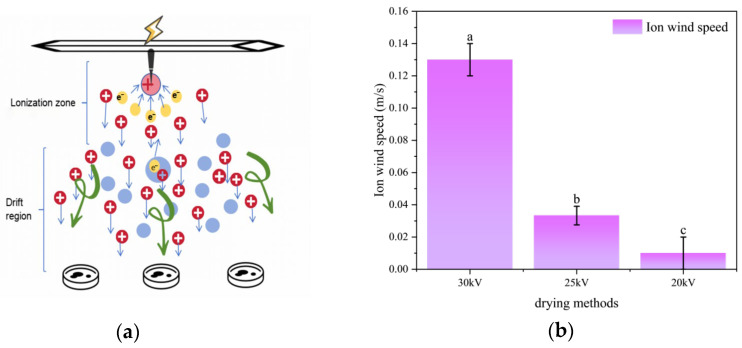
(**a**) Principle of ion wind formation (**b**) Ion wind speed. Different letters indicate significant differences between sample means (*p* < 0.05).

High voltage was applied to the needle-plate electrode through a high-voltage discharge box, causing discharge at the needle tip. The air molecules near the needle tip were ionized, generating a large number of free electrons and positive ions. Under the action of the electric field force, these charged particles accelerated towards the plate electrode and collided with air molecules, leading to the ionization of more air molecules and their directional movement, thus forming an ion wind, as shown in Figure 2a. Figure 2b shows the ion wind speed values generated under different voltages. Different letters above the bars indicate statistically significant differences.Statistical analysis confirmed that the ion wind speed generated under different voltages exhibited significant differences (*p* < 0.05), and its value increases with the increase in voltage [24,25,26,27,28,29,30].

**Figure 3:**
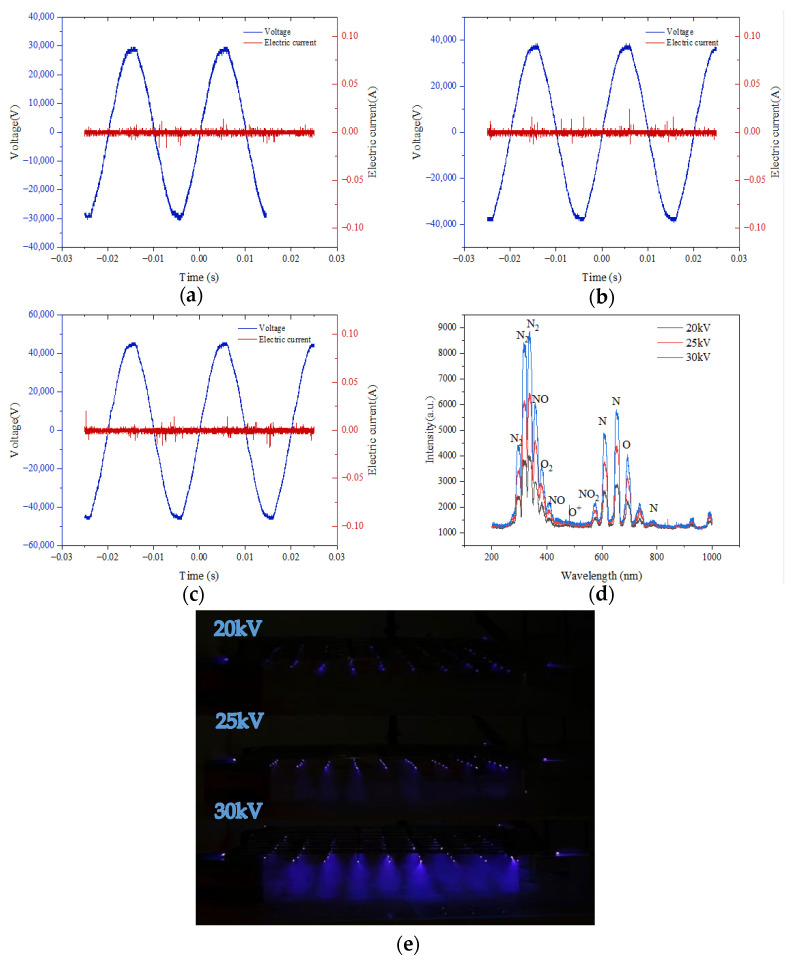
(**a**) Diagram of the 20kV voltage and current waveform; (**b**) 25kV voltage and current waveform diagram; (**c**) 30kV voltage and current waveform diagram; (**d**) emission spectra under different voltages; and (**e**) discharge morphology diagrams under different voltages.

Anukiruthika et al. conducted a systematic analysis of the mechanism and basic concepts of dielectric barrier discharge plasma drying, pointing out that its drying mechanism is driven by the combined effects of ion wind blowing, electric field action, and high-energy particle injection [31]. In Figure 3a–c are the voltage and current waveform diagrams measured under 20 kV, 25 kV, and 30 kV voltages, respectively. The voltage waveforms in the figure are obvious sine waves, with their peaks increasing as the voltage rises, and the frequency is 50 Hz. From the current waveform diagrams, it can be seen that the discharge process belongs to filamentary discharge. The voltage and current waveforms measured by Misra et al. are similar to the results of this study [32].

The plasma generated during the discharge process contains a population of charged particles, predominantly electrons and positive ions, which govern the discharge characteristics. Since the mass of electrons is much smaller than that of ions, the discharge current waveform is mainly determined by the movement behavior of electrons. As the discharge voltage slowly increases, when discharge filaments appear between the electrodes, the voltage amplitude reaches the breakdown voltage. Continue to increase the voltage until the discharge becomes stable, at which point the discharge area uniformly fills the space between the electrodes. As shown in the discharge morphology in Figure 3e, it can be seen that as the input voltage increases, the discharge area gradually expands. It can be clearly observed from the figure that a blue-purple glow is produced during the discharge process. At lower voltages, only a weak light can be observed, while at 30 kV, the blue-purple light is the strongest, indicating that the discharge phenomenon becomes more significant as the voltage increases. At the same time, the uniformity and area of the discharge between the electrodes also gradually expand as the voltage increases.

Figure 3d shows the plasma emission spectra under different voltages. It can be seen from the figure that the active substances produced by the discharge are mainly concentrated in the wavelength ranges of 200–400 nm and 600–800 nm. Among them, the characteristic peaks of N_2_ molecules are near 315 nm, 340 nm, 357 nm, 379 nm, and 393 nm; NO_2_ is near 594 nm; N atoms are near 631 nm, 674 nm, 761 nm, and 811 nm; and O atoms are near 715 nm [26,33,34]. The concentration of various particles beneath the needle tip gradually increased over time [35]. In the plasma substances produced by the discharge in this experimental device, most of the strong peaks correspond to N_2_ molecules. This is because in the discharge process, ground state particles are excited by electron collisions, and the resonance radiation state of N_2_ molecules is higher than that of O_2_ molecules, so N_2_ becomes the main excited molecule. The content of each active substance under different voltages varies significantly, and as the voltage increases, the content of each substance gradually increases, indicating that the electric field enhances the ionization process and generates more active particles. When the voltage reaches 30 kV, the content of active substances reaches the maximum value. Precise regulation of the EHD discharge current allows for direct control of the ion concentration in the discharge space, enabling precise command over the entire discharge dynamics [36].

### 3.2. Analysis of the Drying Characteristics of Astragalus Slices

**Figure 4:**
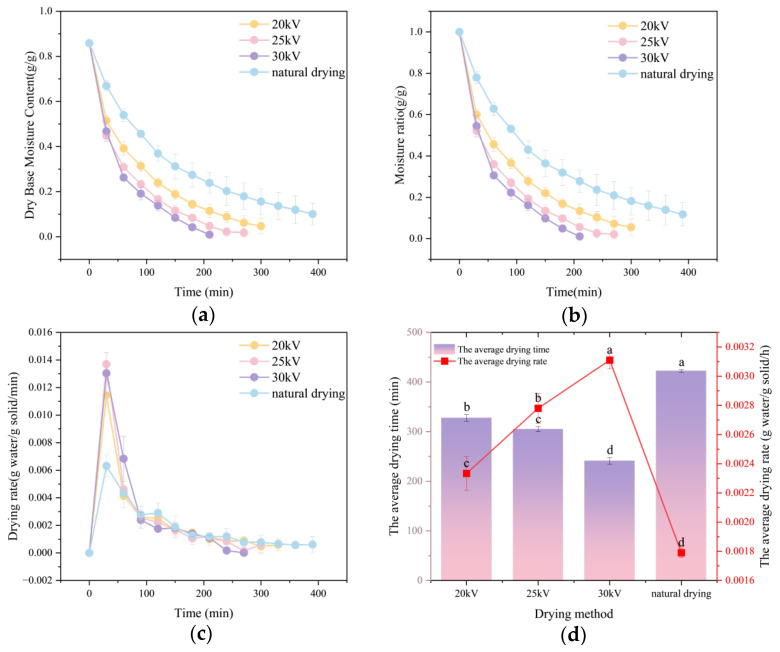
Analysis of the drying characteristics of Astragalus slices. (**a**) Changes in moisture content of Astragalus slices over time. (**b**) Changes in moisture ratio of Astragalus slices over time. (**c**) Changes in drying rate of Astragalus slices over time. (**d**) Changes in average drying time and average drying rate. Different letters indicate significant differences between sample means (*p* < 0.05).

Figure 4a–c depict, respectively, the variations in moisture content, moisture ratio, and drying rate of Astragalus membranaceus slices over time under different drying voltages and natural air-drying conditions. Evidently from the figures, at a drying voltage of 30 kV, both the moisture content and moisture ratio exhibit the steepest decline as the drying time progresses. Although the drying rate attains its peak value at 25 kV, during a certain period subsequent to this peak, the drying rate under a 30 kV voltage remains the highest. In the early stage of drying, the moisture content and moisture ratio decrease rapidly, corresponding to a relatively high drying rate. Nevertheless, as the drying process continues, the rate of decrease in the moisture ratio gradually decelerates, and the drying rate concomitantly diminishes.

This phenomenon can be attributed to the EHD mechanism. In the EHD process, the ion wind exerts a purging effect on the water molecules at the material surface, while the electric field enhances the mobility of water molecules, thus accelerating the drying rate. The drying rate of Astragalus membranaceus slices is primarily governed by the evaporation rate of surface moisture and the diffusion rate of internal moisture. During the initial stage, the combined action of the electric field and ion wind facilitates the rapid evaporation of surface moisture, thereby augmenting the drying rate. However, as the moisture content of the slices gradually decreases, the amount of available surface moisture for evaporation dwindles, which ultimately results in a decline in the drying rate during the later stage.

Figure 4d demonstrates that EHD significantly reduced drying time; with an increase in the drying voltage, the drying time of Astragalus membranaceus slices is remarkably shortened (*p* < 0.01). With 30 kV achieving a 57% reduction versus ND. Moreover, the average EHD drying rates at voltages of 20 kV, 25 kV, and 30 kV are 1.31, 1.46, and 1.73 times the average natural drying rate, respectively. Zhang Li et al. used EHD to dry potatoes [9], and Zhang Jie et al. used EHD to dry yams [37]. They all reached similar conclusions. That is to say, the drying rate of materials increases with the rise in the EHD voltage.

Subsequently, the effective moisture diffusion coefficient (unit: ${\;m}^{2}/s$) is introduced. This coefficient serves to quantify the rate of moisture migration within the Astragalus membranaceus slices. It also mirrors the ease of moisture diffusion from the interior to the surface during the drying process. The coefficient of determination is a measure of the goodness of fit, indicating the extent of agreement between the linear regression model and the actual data. Its value ranges from 0 to 1. A value closer to 1 implies a higher level of reliability of the model.

As presented in Table 1, it is evident that the $D_{eff}\;$ value of increases with an increase in voltage, according to the following order: 30 kV > 25 kV > 20 kV > natural drying. When the EHD voltage parameters are 20 kV, 25 kV, and 30 kV, the values are 5.1, 3.5, and 1.99 times that of natural drying, respectively.

This phenomenon stems from the cell electroporation effect induced by EHD. It destroys the integrity of cell membranes to form microporous channels, significantly improving the permeability of cell membranes. The impact of ionic wind on the biological matrix disrupts the saturated air layer and promotes the diffusion of moisture from the interior to the surface of the matrix, thereby significantly enhancing the evaporation effect and accelerating the transmembrane transport of water. This finding is consistent with the conclusions drawn by Wang et al. in their study on drying carrots using EHD technology [20].

Notably, all R^2^ values exceeded 0.98, indicating a high degree of fit and confirming the reliability of the applied linear model.

### 3.3. Analysis of Rehydration Rate of Processed Astragalus Slices

**Figure 5:**
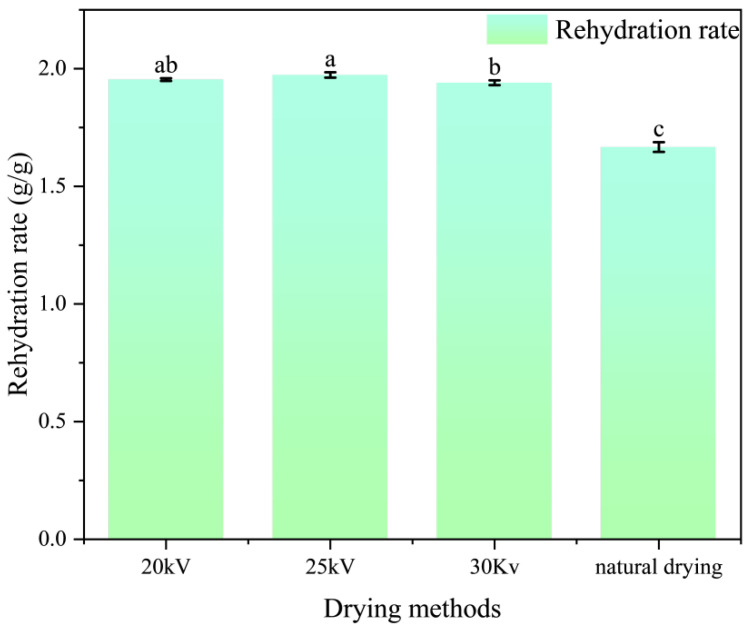
Rehydration rate of astragalus slices under different drying methods. Different letters indicate significant differences between sample means (*p* < 0.05).

The rehydration rate can reflect the tissue structure characteristics of medicinal materials to a certain extent, indicating the ability of the materials to return to their fresh state before drying, after absorbing water [38]. A higher rehydration rate not only facilitates the dissolution of active ingredients but also has a significant impact on quality evaluation, making it an important indicator for assessing the feasibility of drying methods. As shown in Figure 5, the rehydration rate of Astragalus membranaceus slices in the EHD treatment group was significantly higher than that in the natural drying group (*p* < 0.01). Under the voltages of 20 kV, 25 kV, and 30 kV, the rehydration rates were 1.17 times, 1.18 times, and 1.16 times that of the natural drying group, respectively. The rehydration rate of medicinal materials is closely related to the pore size of their matrix microstructure [39]. This result indicates that the EHD drying method more effectively retains the internal pore structure of Astragalus membranaceus slices. This might be due to the fact that EHD increases the drying rate, promoting rapid water migration within the material and thus forming more micro-pores and voids inside the cells. These micro-pore structures remain relatively connected after drying, providing good channels for water penetration during the rehydration process.

The influence of different voltage parameters on rehydration performance was not significantly different, which is consistent with the conclusion drawn by Anjin X et al. in their study on EHD drying of Lentinula edodes slices [40]. Li Yeqing et al. compared the rehydration characteristics of chinaberry peaches subjected to different drying methods. Their findings indicate that the rehydration ratio was highest for hot air-dried samples, followed by vacuum-dried samples, with naturally dried samples exhibiting the lowest ratio. Except for hot air drying at 60 °C, the rehydration ratios of chinaberry peaches dried under the same method but with different parameters were comparable [41]. This further demonstrates that, within a certain range, the drying method significantly influences the material’s rehydration ratio, while drying parameters exhibit no strong correlation. Polat Ahmet et al. compared the effects of EHD, EHD-HAD, and HAD on the drying of ginger and found that the ginger samples dried by EHD exhibited better original appearance and microstructure [42]. Compared to hot drying methods, EHD significantly promotes food rehydration, reflecting its relatively minor impact on material structure and quality. Guo, M.H. et al. also obtained dried products with better rehydration properties by using EHD to dry alfalfa [43].

### 3.4. Analysis of the Shrinkage Rate of Astragalus Slices

**Figure 6:**
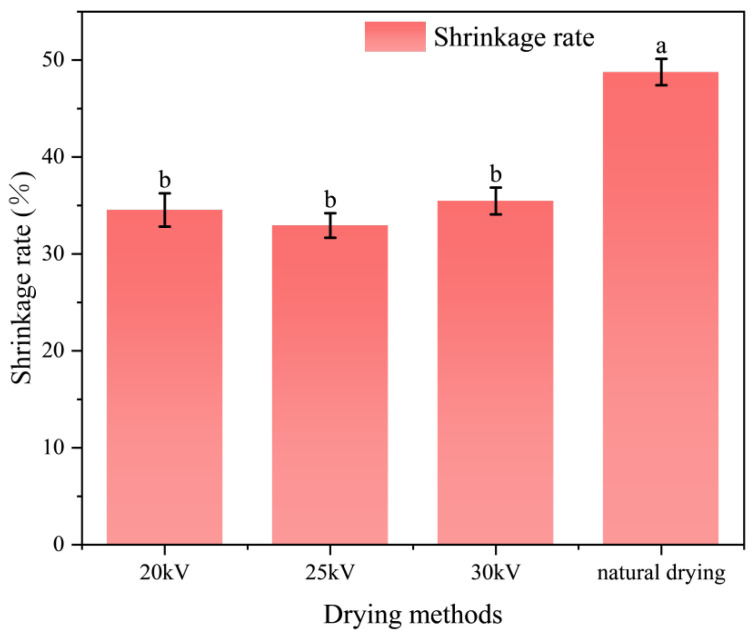
Contraction rate of astragalus slices under different drying methods. Different letters indicate significant differences between sample means (*p* < 0.05).

Shrinkage rate is an important indicator for evaluating the drying process, which can reflect the impact of drying technology on the microstructure of materials and further assess the stability and quality of material drying [44]. As shown in Figure 6, under the voltage conditions of 20 kV, 25 kV, and 30 kV, the shrinkage rate of EHD drying was significantly lower than that of natural drying (ND) (*p* < 0.05), and it showed a trend of “first decreasing and then increasing”. The optimal value was reached at 25 kV. The shrinkage rates of the processed astragalus slices after drying at 20 kv, 25 kv, and 30 kv EHD were 0.71, 0.68, and 0.73 times that of the naturally dried slices, respectively. This phenomenon can be attributed to the slow water migration during natural drying, resulting in a large water gradient, which causes the tissue to be exposed to shrinkage stress for a long time, thus forming a dense structure or even cracks. In contrast, EHD drying accelerates surface water evaporation through ionic wind and is carried out at a lower temperature, reducing thermal stress and helping to maintain the porosity and looseness of the cell structure, thereby effectively inhibiting shrinkage. As the voltage increases, the ionic wind speed increases, water migration accelerates, and the drying time is significantly shortened, thereby reducing the exposure time of Astragalus membranaceus slices to shrinkage stress. However, when the voltage is further increased, it may exceed the tolerance threshold of Astragalus membranaceus slices, causing cell damage and destruction of the cell wall skeleton, which leads to a slight increase in the shrinkage rate. Nevertheless, its shrinkage rate is still significantly better than that of natural drying. There was no significant difference in the contraction rate under different voltages (*p* > 0.05).

Xu Yan et al. found that the contraction rate of scallops after EHD drying was lower than that of ND [45]. Dutta et al. discovered that pre-treatment with an electric field could improve the quality of dried mushrooms and reduce the contraction rate. This further verified the conclusion of our experiment [46].

### 3.5. Analysis of the Surface Color of Dried Astragalus Slices

The change in color directly affects the sales volume of the product [47]. During the drying process of Astragalus membranaceus slices, various chemical reactions such as Maillard reaction, pigment degradation, and enzymatic browning occur, which cause changes in their surface color. “Judging quality by appearance” is a unique quality evaluation method for Chinese medicinal materials and decoction pieces in the traditional Chinese medicine system. “Appearance” refers to the external characteristics, including shape, color, and odor; “Quality” refers to the authenticity and superiority of the internal properties. Among them, color is the core element of “judging by appearance”, covering surface color, internal color, and color changes before and after processing [48]. Therefore, “judging by appearance” plays an important role in the identification of Chinese medicinal materials and decoction pieces.

**Figure 7:**
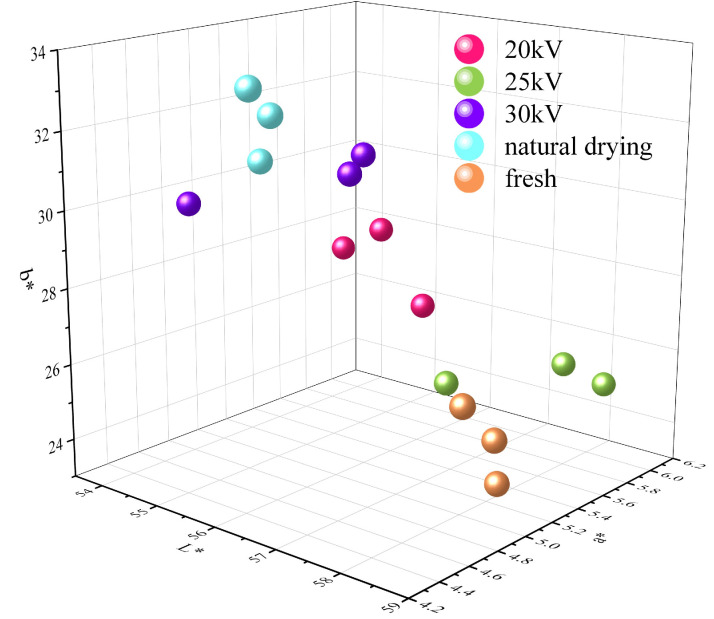
Distribution map of the surface color of Astragalus slices after drying by different methods.

As shown in Figure 7 and Table 2, the yellowness value of Astragalus membranaceus slices after drying is ranked as 25 kV < 20 kV < 30 kV < ND, the redness value is ranked as ND < 30 kV < 20 kV < 25 kV, and the lightness value is ranked as 20 kV < 30 kV < ND < 25 kV. When the voltage is 25 kV, the color of the samples in the EHD treatment group is the closest to that of the fresh samples, indicating that the color and quality of the Astragalus membranaceus slices in this treatment group are superior. The statistical significance of the differences in all color parameters among the drying treatments is presented in Table 2.

During the drying process, the phenolic compounds in Astragalus membranaceus slices are prone to react with oxygen and polyphenol oxidase to form quinone substances, which further polymerize to form black-brown precipitates and eventually degrade into melanin and other orthoquinone compounds. In addition, the phenolic hydroxyl groups in flavonoids are also easily oxidized to form quinone substances, thereby affecting their surface color [49]. The color of the 25 kV treatment group was the best, indicating that under this voltage condition, the oxidation reaction was effectively inhibited. Browning is a common color change phenomenon in natural foods, mainly including enzymatic browning and non-enzymatic browning. Traditional Chinese medicinal materials are rich in water, enzymes, and polyphenols, so they are prone to browning during the drying process. When browning occurs in traditional Chinese medicinal materials such as Astragalus membranaceus, it is often accompanied by color changes, the production of off-flavors, and the loss or transformation of active ingredients, thereby affecting their quality and commercial value [50].

**Figure 8:**
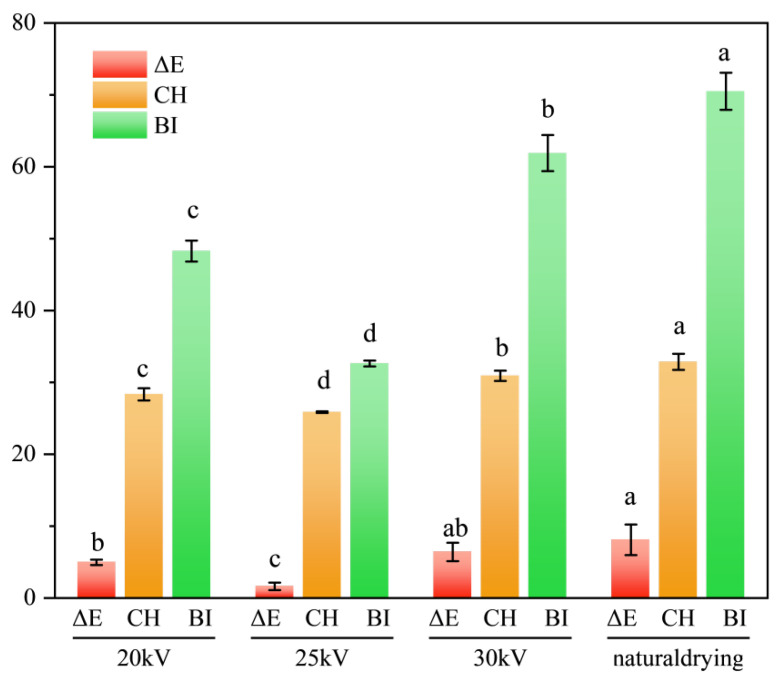
Changes in surface color values ΔE, color saturation CH, and browning index BI of Astragalus slices after drying by different methods. Different letters indicate significant differences between sample means (*p* < 0.05).

The reduction in BI indicates that EHD treatment can effectively inhibit the oxidation reaction of Astragalus membranaceus slices, thereby reducing surface browning, among which the 25 kV treatment effect was the best and the BI value was only 0.46 times that of the ND treatment group. As shown in Figure 8, compared with the ND treatment group, the surface color change value ΔE, color saturation CH, and browning index BI of the EHD treatment group were significantly reduced (*p* < 0.05) and reached the minimum value at 25 kV. The smaller the ΔE, the closer the color of the dried sample is to that of the fresh sample. The ΔE of the 25 kV EHD treatment group was only 0.13 times that of the ND treatment group.

Lin Jiayao et al. also found in her research on the electric field treatment of Pleurotus eryngii that the electric field could effectively inhibit browning, further supporting the conclusions of this study [51]. In addition, Bai et al. found that for seaweed, EHD was more effective than oven drying in maintaining the natural color of the seaweed [52]. The color saturation CH depends on the redness value and yellowness value on the surface of the sample. For Astragalus membranaceus slices, the yellowness value is dominant, so the CH value of the ND treatment group is the highest.

In conclusion, the 25 kV EHD treatment group performed best in terms of “high brightness, balanced red and yellow, and close to natural color”, achieving the optimal color effect.

### 3.6. Analysis of Nutritional Components in Astragalus Root Slices

The nutritional components are the basis of the pharmacological effects of Astragalus membranaceus. Studying the retention of these components can effectively prevent the reduction in efficacy due to improper drying parameters. Astragaloside IV, as the core component of saponins, is one of the legal quality evaluation standards for Astragalus membranaceus in the Chinese Pharmacopeia (2020 Edition), directly reflecting the quality of the medicinal material. The representative flavonoid active substance calycosin is listed as a legal content control index for Astragalus membranaceus in the Chinese Pharmacopeia and is related to the stability of pharmacological effects [53]. Although polysaccharides are not included in the pharmacopeia standards, as one of the most abundant active components in Astragalus membranaceus, they have core functions such as immune regulation and have been identified as quality markers (Q-Markers) in research, which are crucial for nutritional evaluation [54]. Therefore, this study comprehensively considers the requirements of legal standards and functional nutritional evaluation and selects these three nutritional components as evaluation indicators.

**Figure 9:**
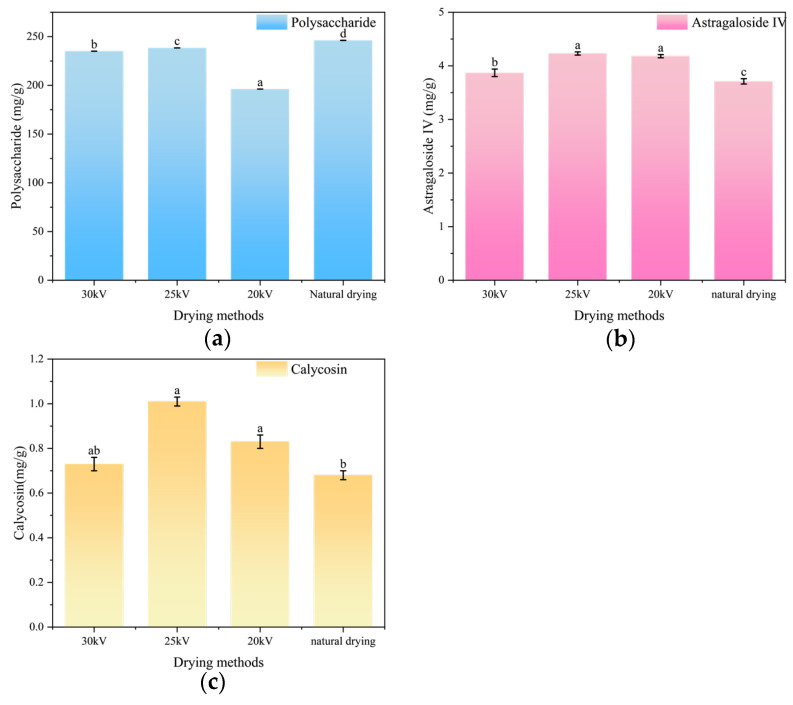
Nutritional components of Astragalus root slices under different drying methods. (**a**) Polysaccharide retention. (**b**) Astragaloside IV retention. (**c**) Calycosin retention. Different letters indicate significant differences between sample means (*p* < 0.05).

As shown in Figure 9a–c, respectively, present the contents of polysaccharides, astragaloside IV, and calycosin in Astragalus membranaceus slices after different drying methods. The EHD group had a relatively lower polysaccharide content than the control group, as shown in Figure 9a. Anjin X et al. proposed the “crust formation phenomenon” when drying mushroom slices using the EHD technology [40]. This phenomenon might be caused by the contraction and rupture of mycelial tubes during rapid drying, leading to the migration of intracellular soluble solids (such as sugars, minerals, and vitamins) along with water to the sample surface. The high-voltage electric field can intensify the electroporation effect on cell membranes, promoting the exudation of intracellular substances. These exudates deposit on the sample surface, eventually forming a crust layer. Unlike mushroom polysaccharides, astragalus polysaccharides are more sensitive to oxidation and have a looser structure, making them prone to polarization imbalance in an electric field. Polysaccharides degrade in the crust layer, leading to greater polysaccharide loss. In contrast, the control group used natural drying, which is air-drying in the shade. This method avoids the negative impact of light and high temperature on polysaccharide content, resulting in a relatively higher polysaccharide content in the control group.

The results in Figure 9b,c indicate that the contents of astragaloside IV and calycosin in dried Astragalus membranaceus slices follow the following order: 25 kV > 20 kV > 30 kV > natural drying. Astragaloside IV belongs to the category of saponins and possesses various biological activities. During the drying process of Chinese medicinal materials, the loss of saponin components is mainly due to enzymatic degradation and high-temperature degradation [55]. The astragaloside IV content in samples treated by EHD was significantly higher than that in the control group (*p* < 0.05). This phenomenon may be related to the fact that EHD drying significantly shortens the drying time, thereby reducing the duration that nutrients are exposed to high humidity and undergo oxidation, enzymatic hydrolysis, and other reactions. Additionally, the high-voltage electric field may inhibit the activity of some enzymes that degrade astragaloside IV and calycosin. Chen et al.’s research has shown that high-voltage electrostatic fields have a positive effect on inhibiting the activity of related degrading enzymes in blueberries [56]. EHD is more conducive to the retention of astragaloside IV than ND, which is consistent with the research results of Qianrui Hou et al. [12].

However, the electric field discharge process can produce ozone, and the ozone concentration and its generation rate increase with the increase in the applied voltage [57]. It is worth noting that ozone can oxidize astragaloside IV [58]. Meanwhile, as a type of flavonoid compound, chrysophanol has pharmacological effects such as anti-inflammatory and antioxidant properties [59]. It is highly susceptible to oxidation and decomposition under the influence of light, heat, and oxygen. Therefore, when the voltage is too high, it may trigger unstable discharge phenomena, thereby accelerating the decomposition of calycosin. Alireza Mousakha et al. also found that when thawing tuna using a high-voltage electrostatic field, tuna chunks oxidized faster under high electrostatic field intensity than under low electrostatic field intensity [60].

In conclusion, both astragaloside and calycosin reached their maximum concentrations at 25 kV treatment conditions, measuring 1.14 times and 1.38 times higher than those in the naturally dried group, respectively. This indicates that the electric field drying voltage (EHD) should not be excessively high but rather maintained within an appropriate range. Notably, Wang et al. observed in their study of electric field treatment of oat hay that nutrient content initially increased with rising drying voltage before showing a subsequent decrease, a trend consistent with our findings [61]. However, EHD drying was not conducive to the retention of astragalus polysaccharides. The best retention of astragalus polysaccharides in the EHD group was the 25 kV treatment group, which was 0.97 times that of the natural drying group.

### 3.7. Correlation Results of Determined Parameters

**Figure 10:**
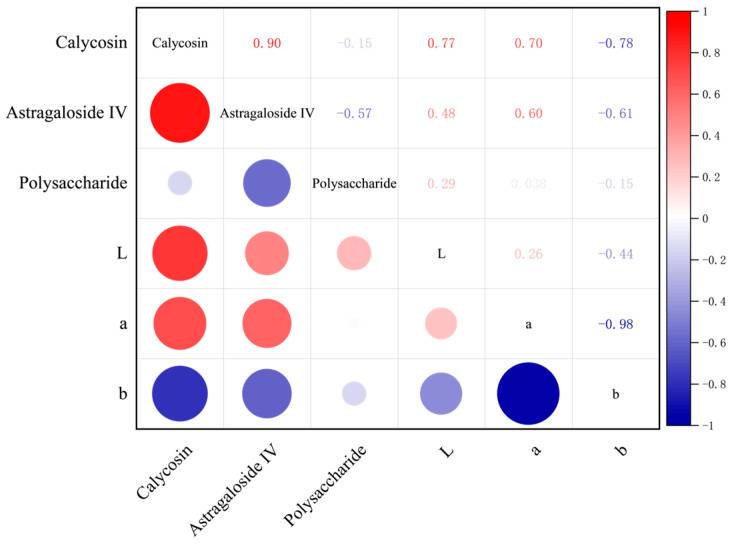
Correlation coefficient matrix between the surface color of astragalus slices and the content of nutrients.

The surface color of Astragalus membranaceus slices is influenced by multiple factors during the drying process, such as temperature and humidity. The pigment changes, to a certain extent, can reflect the retention of internal nutrients. As shown in Figure 10, according to the correlation heatmap analysis, calycosin is the main driver of color changes, while astragaloside IV plays a secondary leading role. The L value and a value are positively correlated with the content of calycosin and astragaloside IV, while the b value is negatively correlated.

**Figure 11:**
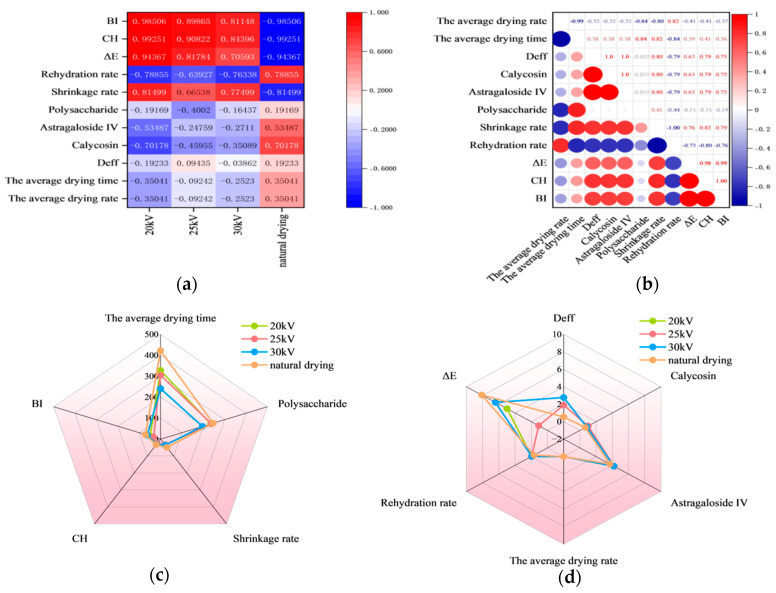
(**a**) Heat map showing the correlation between different drying methods and the drying index of astragalus slices. (**b**) Pearson correlation coefficient matrix. (**c**,**d**) Radar charts.

To comprehensively evaluate the effects of different drying methods on the quality of Astragalus membranaceus slices, correlation analysis and one-way analysis of variance (ANOVA) were performed. ANOVA confirmed the drying method significantly influenced all parameters (*p* < 0.05). To more intuitively illustrate the influence of different drying methods on the drying indicators of Astragalus membranaceus slices, we normalized the experimental data and conducted a correlation analysis, thereby obtaining the correlation coefficients between different drying methods and drying indicators. Figure 11a shows that under the effect of EHD, BI, CH, and E exhibit a high degree of correlation, indicating that EHD can effectively inhibit the browning reaction of Astragalus membranaceus slices during the drying process. This is supported by significant differences in ΔE and BI (*p* < 0.01). In addition, the EHD drying method can significantly increase the rehydration rate, reduce the shrinkage rate, and accelerate the drying rate, thereby shortening the drying time and being more conducive to the retention of nutrients. ANOVA confirmed these quality improvements were significant (*p* < 0.01).

Figure 11b presents the Pearson correlation coefficients among the various detection indicators. The results show that the effective moisture diffusion coefficient is significantly positively correlated with the retention rates of calycosin and astragaloside IV as well as the color indicators, indicating that the larger the effective moisture diffusion coefficient, the better the retention effect of these two active components and the better the surface color. These key parameters were all significantly affected by the drying method (*p* < 0.05). Additionally, the drying rate is significantly positively correlated with the rehydration rate and significantly negatively correlated with the shrinkage rate, suggesting that increasing the drying rate helps improve the rehydration performance and reduce shrinkage.

Figure 11c,d, respectively, present the radar charts of each treatment group. From the area enclosed by the radar charts, it can be seen that, considering the drying rate and various drying quality indicators comprehensively, the EHD drying method under 25 kV voltage has the best overall performance and is the most suitable drying method for Astragalus membranaceus slices. The superior performance of the 25 kV treatment is a direct result of its significant positive effects on multiple quality parameters. 

## 4. Conclusions

In this study, it was found that compared with the control group (ND), the EHD drying group significantly shortened the drying time and increased the effective water diffusion coefficient, the drying rate through the ion wind sweeping effect, and the injection of high-energy particles. At the same time, the shrinkage rate decreased, the rehydration rate increased, and the surface color was closer to the fresh state, indicating that EHD caused less damage to the cell structure of Astragalus. In addition, EHD drying helped to retain astragaloside and calycosin to a certain extent. However, this method had a certain negative impact on the content of Astragalus polysaccharides. In conclusion, EHD drying technology shows a good application prospect in the processing of Chinese medicinal materials.

In the subsequent research, we will attempt to combine EHD with other pre-treatment techniques to explore their effects on astragalus or other Chinese medicinal herbs. We hope this will lay a foundation for the wider application of EHD technology.

## Figures and Tables

**Table 1:** Effective moisture diffusion coefficients inside Astragalus slices under different drying methods. Different superscript letters within the same column indicate significant differences (*p* < 0.05).

Drying Methods	Linear Model	R^2^	k_0_ (s^−1^)	$D_{eff}$(m^2^/s)
20 kV	$lnM_{R}=-1.19\times 10^{-4}t-0.268$	0.995	1.91 × 10^−4^	$1.08\times 10^{-10}$
25 kV	$lnM_{R}=-2.09\times 10^{-4}t-0.100$	0.987	2.09 × 10^−4^	$1.90\times 10^{-10}$
30 kV	$lnM_{R}=-3.05\times 10^{-4}t+0.135$	0.983	3.05 × 10^−4^	$2.77\times 10^{-10}$
natural drying	$lnM_{R}=-5.97\times 10^{-5}t-0.543$	0.994	5.97 × 10^−5^	$5.42\times 10^{-11}$

**Table 2:** Color changes in Astragalus root slice before and after drying. Different letters indicate significant differences between sample means (*p* < 0.05).

Drying Methods	L*	a*	b*	∆E	CH	BI
fresh	58.29 ± 0.04	5.04 ± 0.14	24.92 ± 1.22	-	25.42 ± 1.17	-
20 kV	54.28 ± 0.34 ^c^	5.59 ± 0.04 ^b^	27.79 ± 0.85 ^c^	4.98 ± 0.12 ^c^	28.34 ± 0.18 ^c^	48.4 ± 0.6 ^c^
25 kV	57.59 ± 0.60 ^a^	5.80 ± 0.34 ^a^	25.20 ± 0.28 ^d^	1.08 ± 0.30 ^d^	25.86 ± 0.15 ^d^	32.7 ± 0.5 ^d^
30 kV	55.19 ± 0.96 ^b^	5.09 ± 0.26 ^c^	30.49 ± 0.65 ^b^	6.42 ± 0.18 ^b^	30.91 ± 0.21 ^b^	61.9 ± 0.7 ^b^
natural drying	55.79 ± 0.43 ^b^	4.60 ± 0.20 ^d^	32.49 ± 1.16 ^a^	8.10 ± 0.25 ^a^	32.86 ± 0.32 ^a^	70.5 ± 0.8 ^a^

## Data Availability

The original contributions presented in this study are included in the article. Further inquiries can be directed to the corresponding author.
